# Metastasis on pause: How dormant tumor cells stay hidden within the tumor microenvironment and evade immune surveillance

**DOI:** 10.1002/1878-0261.70259

**Published:** 2026-04-17

**Authors:** Kanishka Tiwary, Jose Javier Bravo‐Cordero

**Affiliations:** ^1^ Division of Hematology and Oncology, Department of Medicine The Tisch Cancer Institute, Icahn School of Medicine at Mount Sinai New York New York USA

**Keywords:** disseminated tumor cells, immune evasion, metastatic niche, quiescence, tumor dormancy, tumor microenvironment

## Abstract

Metastasis remains the leading cause of cancer‐related mortality. Even after major advances in early detection and systemic therapies, long‐term disease recurrence frequently arises from the presence of dormant disseminated tumor cells (DTCs) at distant sites. Dormant DTCs disseminate from the primary tumor and reside in secondary organs in a reversible quiescent state characterized by minimal proliferation, enabling resistance to therapies that target actively dividing cells. Despite their inactivity, dormant DTCs are far from inert. Dormant DTCs dynamically interact with the surrounding tumor microenvironment (TME), including stromal, vascular, and immune components, to establish niches that maintain quiescence while limiting immune detection. While the mechanisms by which proliferating cancer cells evade immune surveillance have been extensively studied, the processes governing immune regulation, immune‐mediated dormancy, and immune evasion of dormant DTCs remain incompletely integrated across literature. In this review, we explore recent advances describing how microenvironmental cues and immune pressures converge on tumor cell–intrinsic programs to sustain dormancy, promote immune tolerance, and enable long‐term survival of DTCs across different organs and cancer types. We further discuss conditions that disrupt this equilibrium and drive escape from dormancy, as well as emerging therapeutic strategies aimed at eliminating or controlling dormant DTCs by targeting dormancy‐specific immune and microenvironmental interactions.

## Graphical abstract

Dormant cancer cells can hide in distant organs for years, evading treatment and the immune system. This review highlights how signals from the surrounding tissue and immune environment keep these cells inactive or trigger their reawakening. Understanding these mechanisms may help develop therapies to eliminate or control dormant cells and prevent cancer relapse.

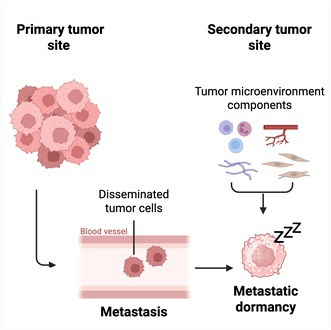

**Abbreviations**
ATRA: all‐trans retinoic acid
AZA: 5‐azacitidine
BACH1: BTB and CNC homology 1
BHLHE41: basic helix–loop–helix family member e41
BRD7: bromodomain containing 7
CAR T cells: chimeric antigen receptor T cells
CD8^+^ T cells: cluster of differentiation 8 positive T cells
CDKN1A (p21): cyclin‐dependent kinase inhibitor 1A
CDKN1B (p27): cyclin‐dependent kinase inhibitor 1B
CGRP: calcitonin gene‐related peptide
COL3A1: collagen type III alpha 1 chain
COX‐2: cyclooxygenase‐2
CPT‐11: irinotecan
CSCs: cancer stem cells
DDR1: discoidin domain receptor 1
DKK3: Dickkopf WNT signaling pathway inhibitor 3
DTCs: disseminated tumor cells
ECM: extracellular matrix
EGFR: epidermal growth factor receptor
ERK: extracellular signal‐regulated kinase
ER stress: endoplasmic reticulum stress
FAK: focal adhesion kinase
FGFR2: fibroblast growth factor receptor 2
FOXM1: forkhead box M1
FoxP3^+^ T cells: forkhead box P3 positive regulatory T cells
G0/G1: G0/G1 phase of cell cycle
GAS6: growth arrest‐specific 6
GDF10: growth differentiation factor 10
HPA axis: hypothalamic–pituitary–adrenal axis
IFN‐γ: interferon gamma
ISR: integrated stress response
JUN: jun proto‐oncogene
Ki67: marker of proliferation Ki‐67
MAPK: mitogen‐activated protein kinase
MHC I: major histocompatibility complex class I
miRNA/miR: MicroRNA
mTOR: mechanistic target of rapamycin
NETs: neutrophil extracellular traps
NK cells: natural killer cells
NR2F1: nuclear receptor subfamily 2 group F member 1
NRF2: nuclear factor erythroid 2–related factor 2
NSCLC: non–small cell lung cancer
P4HA2: prolyl 4‐hydroxylase subunit Alpha 2
PBAF: polybromo‐associated BAF chromatin remodeling complex
PCNA: proliferating cell nuclear antigen
PD‐1: programmed cell death protein 1
PDAC: pancreatic ductal adenocarcinoma
PERK: protein kinase RNA‐like endoplasmic reticulum kinase
ROCK: Rho‐associated protein kinase
SOX2: SRY‐box transcription factor 2
Src: proto‐oncogene tyrosine‐protein kinase Src
STING: stimulator of interferon genes
TAM receptors: Tyro3, Axl, Mer receptors
TEAD: TEA domain transcription factor
TGF‐β2: transforming growth factor beta 2
TLR7: toll‐like receptor 7
TME: tumor microenvironment
TRPA1: transient receptor potential ankyrin 1
TSP‐1: thrombospondin‐1
ULBP: UL16 binding protein
ULK1: Unc‐51 like autophagy activating kinase 1
uPAR: urokinase plasminogen activator receptor
YAP: yes‐associated protein

## 1 Introduction

In an era of tremendous advancements in cancer treatment modalities, the risk of metastatic relapse for cancer patients remains a significant clinical challenge [1]. Metastasis is a complex, multistep process. Tumor cells leave the primary site of growth, invading the stroma, intravasate into the circulation, and acquire transitional cellular states. Once they overcome various immune and extracellular matrix barriers, they extravasate to seed secondary organs [2]. Upon arrival at secondary sites, disseminated tumor cells (DTCs) can enter a quiescent state and persist for prolonged periods while awaiting microenvironmental conditions permissive for re‐initiation of proliferation and metastatic outgrowth [3]. Indeed, DTCs can remain in this ‘sleeping’ or dormant state in the metastatic niche from years to even decades after initial treatment of primary tumors.

Metastatic dormancy can be broadly described as an arrest in tumor growth where DTCs can stay in an interrupted proliferation state [3]. The mechanisms through which metastatic dormancy is regulated have been historically defined by two principal frameworks, cellular dormancy, and tumor mass dormancy [4]. Cellular dormancy refers to a state in which individual DTCs undergo a reversible growth arrest and is characterized by entry into quiescence. Experimental studies have demonstrated that solitary tumor cells can be maintained in a dormant state through signaling imbalances such as reduced ERK activity relative to p38 MAPK, enforcing cell‐cycle arrest while preserving long‐term cellular viability [5, 6]. In contrast, tumor mass dormancy describes a condition in which small micrometastatic lesions are maintained at a stable size through a dynamic equilibrium between tumor cell proliferation and apoptosis. In this regard, studies have shown that the balance is often dictated by microenvironmental constraints in preventing net tumor expansion despite ongoing cell turnover [7, 8].

More recently, metastatic dormancy has been recognized as a multifaceted and dynamic phenomenon that encompasses additional regulatory layers. Immune‐mediated dormancy arises when immune surveillance constrains tumor outgrowth. For example, experimental tumor model studies in lymphoma and breast cancer have shown that cytotoxic CD8^+^ T cells and interferon‐γ signaling restrain metastatic outgrowth and enforce a state of equilibrium between tumor cells and host immunity [9, 10]. On the other hand, angiogenic dormancy is driven by insufficient neo‐vascularization. This has been supported by experimental studies demonstrating that suppression of new vasculature or maintenance of a stable perivascular niche restricts micrometastatic expansion and maintains tumor lesions in a dormant state until angiogenic signaling permits outgrowth [11, 12, 13, 14]. Finally, extracellular matrix (ECM)–mediated dormancy is governed by biochemical and mechanical cues from the metastatic niche where specific ECM compositions enforce quiescence and survival of dormant DTCs. For example, laminin‐rich basement membrane niches have been shown to maintain breast cancer cell dormancy [15, 16] and dormant DTCs have been shown to have enriched collagen gene expression signatures [17, 18]. It is important to note that these distinct forms of dormancy are not mutually exclusive and often coexist within the same metastatic microenvironment.

Disseminated tumor cells can adapt to diverse metastatic niches by engaging multiple, overlapping dormancy programs and persist long‐term. These properties in turn can influence patterns of late metastatic relapse in patients [1, 19]. Dormancy is particularly prominent in cancers such as breast cancer, prostate cancer, and melanoma, where disease recurrence may occur decades after primary tumor removal. In contrast, cancers including pancreatic ductal adenocarcinoma, small‐cell lung cancer, and glioblastoma are characterized by limited dormancy and rapid relapse [20, 21]. This broad temporal spectrum has profound implications for therapeutic strategies, as cancers associated with long latency require sustained monitoring and interventions capable of targeting clinically undetectable disease. Adding to this challenge, dormant DTCs are inherently resistant to most standard therapies and remain difficult to detect in patients, which has, for a long time, limited the effort for direct clinical characterization.

However, recent advances in experimental tumor modeling, single‐cell sequencing, and spatial transcriptomic technologies have enabled high‐resolution profiling of rare tumor cell populations and their surrounding microenvironments within metastatic niches [22, 23]. These advances have led to the identification of several defining hallmarks of dormant DTCs, which include (i) a deep but reversible quiescent state characterized by G0/G1 arrest; (ii) expression of dormancy‐associated transcriptional programs such as *BHLHE41, NR2F1, CDKN1B, CDKN1A, COL3A1, DDR1, P4HA2*; (iii) latent pluripotency, reflecting a partially dedifferentiated, and reversible cell‐state plasticity; (iv) dynamic interactions with and remodeling of the extracellular matrix; and (v) epigenetic and transcriptional reprogramming [24, 25, 26, 27]. As a whole, these features underscore that dormant DTCs are not inert entities but rather actively maintained cellular states.

A growing body of evidence indicates that dormant DTCs rely on continuous interactions with the tumor microenvironment (TME) to sustain quiescence and evade immune elimination. This concept is supported by clinical observations in which organ transplantation from cancer‐free donors with a prior history of cancer resulted in metastatic outgrowth in immunocompromised recipients [28], highlighting the critical role of immune surveillance in restraining dormant disease. The metastatic niche comprises diverse cellular and acellular components, including endothelial cells, cancer‐associated fibroblasts, immune cells, extracellular matrix, and neuroendocrine elements, all of which contribute to the regulation of DTC fate [29, 30].

In this review, we integrate recent discoveries describing how dormant DTCs receive and interpret signals from the tumor microenvironment and immune system to maintain quiescence, evade immunosurveillance, and persist long term in secondary organs. We focus on molecular and cellular mechanisms through which stromal, vascular, and immune components shape dormant cell states, as well as conditions that disrupt this equilibrium and trigger escape from dormancy. We highlight vulnerabilities unique to dormant DTCs by linking tumor‐intrinsic programs with extrinsic, immune‐mediated control and evasion, and discuss emerging therapeutic strategies to prevent metastatic reactivation or eradicate dormant disease.

## 2 Tumor microenvironmental regulation of dormant DTCs

Dormant DTCs enter and sustain quiescence through tumor‐intrinsic programs that enforce cell‐cycle arrest, preserve survival, and maintain long‐term viability [3]. However, dormant DTCs do not persist in isolation but are actively maintained by signals from the metastatic microenvironment. Therefore, tumor‐intrinsic quiescence programs are reinforced by extrinsic cues derived from vascular, stromal, and extracellular matrix components of the metastatic niche. Before examining individual tumor microenvironment components, it is important to recognize that dormancy is first executed at the level of the tumor cell.

### 2.1 Cell‐intrinsic dormancy programs

A defining feature of cellular dormancy is arrest in the G0/G1 phase, mediated by upregulation of cyclin‐dependent kinase inhibitors such as p21 and p27 and concomitant suppression of proliferation markers including Ki67 and PCNA [31, 32, 33]. At the signaling level, this state is governed by a conserved imbalance between stress‐activated and mitogenic pathways, characterized by high p38 MAPK activity relative to ERK signaling [16, 34]. This p38^high^/ERK^low^ ratio regulates transcriptional networks that enforce quiescence, including activation of dormancy‐associated factors such as NR2F1 and DEC2 [33, 35], while suppressing cell‐cycle progression through inhibition of FOXM1‐ and JUN‐dependent programs [36, 37, 38]. Together, these pathways define a core, tumor cell–intrinsic dormancy program that enforces reversible growth arrest and long‐term persistence of solitary DTCs.

### 2.2 Epigenetic mechanisms of dormancy maintenance

**Fig. 1:**
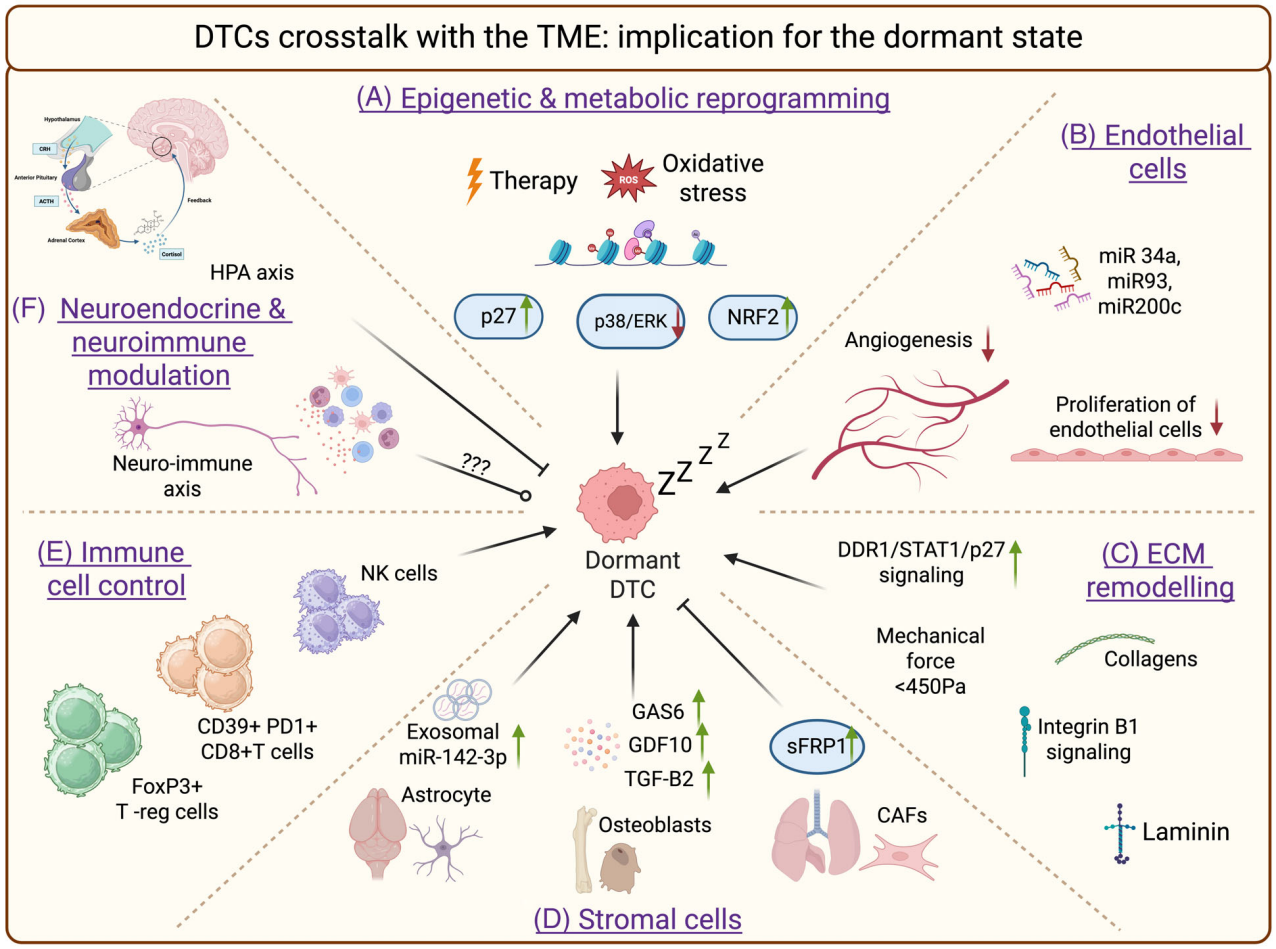
Disseminated tumor cells (DTCs) cross talk with tumor microenvironment (TME): implications for the dormant state. Top clockwise, (A) epigenetic and metabolic reprogramming, (B) endothelial cells, (C) extracellular matrix (ECM) remodeling, (D) stromal cells, (E) immune cells and (F) neuroendocrine and neuroimmune axis role within the TME in dormancy maintenance. Graphical illustrations are created in Biorender.

Looking beyond signaling events, epigenetic reprogramming serves as a critical mechanism for stabilizing intrinsic dormancy programs, particularly in response to therapeutic and microenvironmental stress. In estrogen receptor–positive breast cancer patients receiving neoadjuvant endocrine therapy, it has been demonstrated that treatment can induce durable dormant states through nongenetic mechanisms, enabling dormant phenotype persistence and later awakening without recurrent genetic alterations [39]. At the chromatin level, dormancy has been linked to the activity of chromatin remodeling complexes such as the PBAF complex via BRD7 [40], as well as incorporation of histone variants including macroH2A, which collectively reconfigure chromatin accessibility and reinforce quiescent transcriptional programs [41]. In parallel, metabolic stress adaptation pathways, including NRF2‐mediated antioxidant responses, further support survival of dormant DTCs under hostile conditions [42]. Importantly, these epigenetic and metabolic states reflect both tumor cell–intrinsic plasticity and responsiveness to niche‐derived signals, thereby providing a mechanistic bridge between intrinsic dormancy programs and microenvironmental regulation (Fig. 1A).

### 2.3 Vascular niche‐mediated dormancy signals

Endothelial cells represent a critical cellular component of metastatic niches and play an active role in regulating the fate of disseminated tumor cells (DTCs). Apart from their function as a structural lining of blood vessels, endothelial cells provide paracrine signals that can enforce tumor cell quiescence and maintain long‐term dormancy. Early studies demonstrated that DTCs residing in close association with stable, nonsprouting vasculature adopt a dormant phenotype, whereas disruption of endothelial homeostasis promotes proliferative outgrowth [7, 11, 13, 14]. These findings established the concept of a perivascular dormancy niche, in which endothelial‐derived signals actively restrain tumor cell proliferation.

Mechanistically, endothelial cells maintain dormancy through secretion of quiescence‐inducing factors and suppression of angiogenic signaling. Ghajar et al. demonstrated that endothelial‐derived thrombospondin‐1 (TSP‐1) enforces dormancy of breast cancer DTCs by maintaining quiescent signaling programs within the perivascular niche [13]. Similarly, seminal studies have shown that vascular niches regulate the balance between dormancy and proliferation in leukemia by providing spatially restricted signals that preserve quiescence [43, 44, 45]. Inhibition of endothelial proliferation and angiogenic sprouting stabilizes these dormancy‐enforcing signals, preventing DTC cell‐cycle re‐entry and significantly reducing metastatic outgrowth.

Consistent with this concept, suppression of angiogenesis has been shown to sustain tumor dormancy across multiple cancer types. Experimental inhibition of endothelial proliferation limits neovascularization and constrains metastatic lesions to a dormant state [46, 47]. At the molecular level, noncoding RNAs have emerged as regulators of endothelial‐mediated dormancy. In osteosarcoma models, microRNAs such as miR‐34a, miR‐93, and miR‐200c suppress angiogenic gene expression, thereby limiting vascular expansion and promoting sustained dormancy of tumor cells [48]. Together, these studies highlight endothelial cells as active gatekeepers of metastatic dormancy, capable of enforcing quiescence through angiogenic restraint and perivascular niche signaling (Fig. 1B).

### 2.4 ECM cues in dormant cell maintenance

The extracellular matrix (ECM) represents the acellular structural scaffold of the tumor microenvironment and has long been recognized for its role in providing architectural integrity to tissues. The ECM also actively regulates cell fate decisions through biochemical composition, mechanical properties, and signaling crosstalk with tumor cells. While ECM remodeling is widely implicated in cancer invasion and metastatic progression, accumulating evidence indicates that specific ECM configurations and signaling states play a critical role in enforcing and maintaining dormancy of DTCs (Fig. 1C) [49].

Biomechanical properties of the ECM have been shown to directly influence tumor cell quiescence. In a comprehensive analysis of breast cancer patient datasets and clinical specimens, Li et al. demonstrated that increased ECM stiffness and biomechanical stress can drive cancer cells into a stem‐like quiescent state characterized by cell‐cycle arrest [50]. Specifically, exposure to defined mechanical forces (~450 Pa) induced growth arrest and dormancy‐associated transcriptional programs. The authors further identified an ECM signature enriched in fibrinogen, fibronectin, vitronectin, and elastin that correlated with patient prognosis, highlighting the clinical relevance of ECM‐mediated dormancy regulation.

Cell–ECM adhesion signaling is another critical determinant of dormancy maintenance. β1 integrin has been extensively documented as a central regulator of tumor cell fate decisions, with its suppression being associated with sustained quiescence. Dormant DTCs have been shown to downregulate β1 integrin–dependent signaling pathways, thereby limiting downstream activation of EGFR–ERK signaling and preserving a p38‐dominant stress signaling state [51]. Mechanistically, urokinase plasminogen activator receptor (uPAR), which facilitates integrin β1 and EGFR signaling, is frequently downregulated in dormant cells. Loss of uPAR expression disrupts integrin‐mediated signaling and contributes to the maintenance of growth arrest programs in DTCs [5, 52]. In the brain metastatic niche, astrocyte‐deposited laminin‐211 has been shown to promote dormancy of breast cancer DTCs by sequestering YAP in the cytoplasm, thereby suppressing proliferative transcriptional programs [53].

Multiple ECM components have also been implicated in reinforcing dormancy through direct regulation of tumor cell signaling pathways. Di Martino *et al*. demonstrated that dormant DTCs can establish and maintain a type III collagen–enriched ECM niche that is essential for sustaining quiescence; loss of this collagen III–rich matrix disrupts dormancy‐associated signaling and destabilizes latency [17]. Similarly, studies in breast cancer have identified collagen VII as a marker associated with hypoxia‐induced dormancy, with elevated collagen VII expression correlating with low proliferative indices (Ki67^low^) in patient samples [54]. In colorectal cancer models, collagen XVII has been shown to support dormancy maintenance and therapy resistance by upregulating p21 and p27 and reinforcing a high p38/ERK signaling ratio through inhibition of FAK–YAP signaling [34, 55]. These ECM–collagen–driven signaling programs collectively enforce cell‐cycle arrest and long‐term survival of dormant tumor cells. These studies establish the extracellular matrix as an active regulator of metastatic dormancy rather than a passive structural component. Through biomechanical constraints, integrin‐dependent signaling suppression, and collagen‐mediated transcriptional control, ECM niches impose and stabilize quiescent states in disseminated tumor cells across organ sites. Such ECM‐mediated dormancy programs operate in concert with tumor‐intrinsic and stromal signals to preserve metastatic latency and prevent overt tumor outgrowth.

### 2.5 Stromal cell niches maintain tumor cell quiescence

Disseminated tumor cells (DTCs) can exploit stromal components of organ‐specific microenvironments to actively maintain a dormant, quiescent state. In the bone metastatic niche, osteoblasts have emerged as key regulators of dormancy maintenance. In prostate cancer models, osteoblast‐derived growth differentiation factor 10 (GDF10) and transforming growth factor β2 (TGFβ2) enforce tumor cell quiescence by activating p38 MAPK signaling and suppressing proliferative pathways, thereby restraining metastatic outgrowth and sustaining long‐term dormancy [56]. Similarly, studies in metastasis to the bone marrow have shown that osteoblasts secrete growth arrest‐specific protein 6 (GAS6) and can induce dormancy in prostate cancer and leukemia, through TAM (Tyro3, Axl and Mer) family receptors and downstream MAPK activation [57, 58, 59]. These findings establish the osteoblastic niche as a dormancy‐permissive microenvironment that actively suppresses tumor cell proliferation.

Similarly, in the brain metastatic niche, astrocytes have been shown to exert growth‐inhibitory effects on disseminated tumor cells through paracrine and exosome‐mediated signaling. Astrocyte‐derived exosomes containing microRNA‐142‐3p suppress TRPA1 expression in lung cancer DTCs, leading to reduced FGFR2 signaling and inhibition of cell proliferation, thereby promoting a dormant phenotype [60]. This astrocyte‐mediated signaling axis highlights how stromal cells within the central nervous system contribute to the maintenance of metastatic latency. These studies demonstrate that stromal cells within distinct organ microenvironments actively enforce dormancy through secreted factors, exosomal communication, and niche‐specific signaling programs (Fig. 1D).

## 3 Immune mediated dormancy and immune evasion

Upon extravasation into secondary organs, disseminated tumor cells (DTCs) must adapt to a foreign microenvironment while avoiding elimination by host immune defenses. Multiple immune cell populations, including natural killer (NK) cells, macrophages, neutrophils, and cytotoxic T cells, contribute to immunosurveillance across metastatic niches and represent a critical barrier to metastatic outgrowth. Accumulating evidence indicates that immune pressure can actively restrain DTC proliferation and enforce a state of immune‐mediated dormancy, rather than simply eliminating disseminated cells. In breast cancer models, depletion of NK cells results in increased metastatic burden across multiple organs, highlighting their central role in suppressing early metastatic outgrowth [61, 62, 63]. For example, in the liver metastatic niche, Correia et al. have shown that the interplay between NK cells and hepatic stellate cells acts as a master switch for cancer dormancy in breast cancer. Similarly, Bushnell et al. identified NK cells to be key in the metastatic dormant phenotype in breast cancer. They found that quiescent cancer stem cells (CSCs) were resistant to NK cell cytotoxicity mediated by the expression of BACH1 and SOX2 transcription factors, while proliferative CSCs were sensitive. Whereas Malladi et al. described that dormant DTCs activate SOX‐dependent transcriptional programs to establish a stem‐like state as well as downregulate ULBP ligand for NK cells to evade NK cell‐mediated cytotoxicity. Together, these studies support the concept that immune surveillance is not only cytotoxic but can also maintain disseminated tumor cells in a quiescent, equilibrium‐like state.

Apart from immune surveillance, specific immune cell populations have been shown to actively induce or stabilize dormancy programs in DTCs. In the lung metastatic niche, alveolar macrophages have been reported to maintain breast cancer DTC quiescence through TGF‐β2–dependent signaling, as depletion of alveolar macrophages or TGF‐β receptor loss‐of‐function experiments in DTCs triggered metastatic awakening [64]. In contrast, inflammatory perturbations can disrupt immune‐mediated dormancy. For example, IL‐17A–driven inflammation promotes awakening of dormant breast cancer cells, while interferon‐γ signaling has been shown to directly enforce dormancy programs [65]. These findings underscore that immune‐mediated dormancy is context dependent and dynamically regulated, reflecting the balance between immune restraint and inflammatory cues within metastatic niches.

Dormant DTCs employ multiple strategies to evade immune elimination, many of which are intrinsically linked to their quiescent and solitary nature. As dormant DTCs exist as single cells or rare micrometastatic clusters, their interactions with equally rare antigen‐specific immune cells are limited. Goddard et al. described this phenomenon as ‘relative scarcity’, demonstrating that large physical distances between dormant DTCs and cognate T cells in the lung niche reduce the probability of immune‐mediated killing despite intact antigen recognition [66]. In addition, dormant tumor cells can actively shape an immunosuppressive microenvironment. In breast cancer, dormant DTCs were shown to evade CD8^+^ T cell–mediated immunity by recruiting FoxP3^+^ regulatory T cells through tumor‐derived DKK3, a mechanism linked to a hybrid epithelial–mesenchymal cell state [10]. Immune evasion mechanisms have also been described in other cancer types, including pancreatic ductal adenocarcinoma, where dormant liver DTCs lack MHC class I expression and display unresolved endoplasmic reticulum stress, enabling immune escape and metastatic latency [67]. Conversely, protective immune populations may contribute to dormancy maintenance, as CD39^+^PD‐1^+^CD8^+^ T cells have been associated with restrained metastatic outgrowth and improved control of dormant disease (Fig. 1E) [68].

Interestingly, the ability of immune cells to restrain tumor growth has also been conceptualized within the framework of tumor immunoediting, which includes the phases of elimination, equilibrium, and escape [69]. During the equilibrium phase, immune pressure limits tumor expansion and can maintain residual tumor cells in a dormant state for extended periods. However, sustained immune pressure also selects for tumor cell variants with reduced immunogenicity, including defects in antigen presentation and immune recognition. Dormant DTCs frequently exhibit downregulation of MHC class I molecules, altered antigen processing, or increased expression of immune checkpoint ligands, reducing effective T cell–mediated clearance [70, 71]. This immune sculpting process allows dormant tumor cells to persist under immune control while progressively acquiring immune‐evasive traits that facilitate eventual escape and relapse.

Importantly, immune‐mediated dormancy and awakening are not uniform across tissues but are profoundly shaped by organ‐specific immune landscapes. Distinct immune cell compositions and inflammatory thresholds across metastatic sites have the capacity to dictate whether immune pressure enforces dormancy or facilitates escape. A comparative overview of organ‐specific immune mechanisms regulating dormancy maintenance and metastatic awakening is provided in Table 1, highlighting how immune surveillance, inflammation, and immune evasion differ across metastatic niches.

**Table 1:** Organ‐specific immune regulation of metastatic dormancy and awakening.

Metastatic niche	Cancer type(s)	Dormancy‐maintaining immune cues	Immune‐mediated awakening triggers	Key immune cell populations	Representative references
Lung	Breast cancer, melanoma	NK cell–mediated immune pressure; IFN‐γ–driven quiescence; alveolar macrophage–derived TGF‐β2 enforcing dormancy	IL‐17A–driven inflammation; neutrophil infiltration; NET‐mediated ECM remodeling	NK cells, CD8^+^ T cells, alveolar macrophages, neutrophils	Malladi, Cell, 2016; Albrengues, Nature, 2018 Pereira, Cell Reports, 2025
Bone marrow	Breast cancer, prostate cancer, melanoma	Immune equilibrium maintaining latency; NK and CD8^+^ T cell surveillance	Myeloid skewing; inflammatory cytokines disrupting immune equilibrium	NK cells, CD8^+^ T cells, myeloid cells	Koebel, Nature, 2007; Eyles, J Clin Invest, 2010; Crist & Ghajar, Annu Rev Cancer Biol, 2021
Liver	Pancreatic ductal adenocarcinoma, colorectal cancer	Partial immune restraint within tolerogenic liver environment	Loss of MHC classI; unresolved ER stress enabling immune escape	CD8^+^ T cells, Kupffer cells	Pommier, Nature, 2021; Correia, Nat Rev Immunol, 2023
Brain	Breast cancer, lung cancer, melanoma	Immune privilege limiting cytotoxic clearance; microglia‐mediated restraint	Neuroinflammation; disruption of immune–stromal balance	Microglia, limited T cell infiltration	Sevenich, Nat Rev Cancer, 2018; de Visser & Joyce, Cancer Cell, 2023
Multiple organs	Breast cancer, melanoma, lymphoma	Immune equilibrium phase maintaining latency across organs	Immune exhaustion; checkpoint upregulation; selection of low‐immunogenic variants	CD8^+^ T cells, Tregs, myeloid cells	Koebel, Nature, 2007; Schreiber, Science, 2011; Tallon de Lara, Nat Immunol, 2021

## 4 Escape from dormancy and metastatic reactivation

Metastatic dormancy is a reversible state, and perturbations within the metastatic microenvironment can destabilize dormancy‐maintaining programs and initiate tumor cell reactivation. One of the mechanisms of dormancy escape is the angiogenic switch, whereby previously avascular or poorly vascularized micrometastatic lesions transition to a pro‐angiogenic state that supports tumor expansion. Seminal work by Holmgren et al. demonstrated that suppression of angiogenesis constrains micrometastases in a dormant state, whereas neovascularization permits rapid outgrowth [8]. Subsequent studies revealed that stable perivascular niches actively enforce quiescence, while endothelial activation and vascular remodeling promote metastatic awakening [13]. In parallel, extracellular matrix (ECM) remodeling—including collagen deposition, matrix stiffening, and fibrosis—has been shown to disrupt quiescent signaling and promote re‐entry into the cell cycle through integrin‐ and mechano‐transduction‐dependent pathways [72, 73]. Age‐associated remodeling of metastatic niches, characterized by chronic inflammation and altered ECM composition and cancer associated fibroblasts, further lowers the threshold for dormancy escape, linking tissue aging to increased metastatic risk [74, 75] (Fig. 1D).

Loss of immune‐mediated control represents another critical route through which dormant DTCs escape quiescence. While immune surveillance can maintain DTCs in a state of equilibrium for prolonged periods, inflammatory cues or immunosuppressive shifts can disrupt this balance and trigger metastatic reactivation. Experimental models have shown that depletion of cytotoxic immune populations or impairment of interferon signaling leads to rapid outgrowth from previously dormant lesions [70, 71]. Pro‐inflammatory cytokines have also been implicated as direct awakening signals; for example, IL‐17A–driven inflammation has been shown to override dormancy‐enforcing immune programs and promote metastatic outgrowth in breast cancer models [65]. In addition, neutrophil extracellular traps (NETs), generated during inflammatory responses, remodel the ECM and expose integrin‐binding sites that stimulate proliferation of dormant tumor cells [76]. Taken together, these findings highlight that immune‐mediated dormancy is a fragile equilibrium that can be disrupted by inflammatory or immunosuppressive conditions within the metastatic niche.

Beyond local niche remodeling, systemic host‐derived signals—including neuroendocrine stress responses—can play a pivotal role in triggering escape from metastatic dormancy. Activation of the hypothalamic–pituitary–adrenal (HPA) axis and sympathetic nervous system during chronic stress results in sustained release of glucocorticoids and catecholamines, which suppress antitumor immunity and promote pro‐metastatic niche remodeling [77, 78, 79]. Seminal studies demonstrated that chronic stress enhances metastatic progression through β‐adrenergic signaling, in part by impairing immune surveillance and promoting inflammatory pathways [79, 80].

Adverse social and environmental stressors have been shown to actively condition metastatic target organs toward permissiveness. In this regard, He et al. demonstrated that chronic stress establishes a pro‐metastatic lung niche in breast cancer, characterized by increased fibronectin deposition and neutrophil infiltration, and that depletion of neutrophils significantly attenuated stress‐induced metastatic outgrowth [81]. These findings link stress‐induced inflammation and ECM remodeling directly to dormancy escape. In contrast, neurogenic inputs can also exert protective effects: Futoh et al. reported that vagal nerve signaling suppresses peritoneal metastasis in gastric cancer, highlighting the bidirectional and context‐dependent roles of neural circuits in metastatic regulation [82].

Peripheral neurons have additionally emerged as critical modulators of immune and inflammatory responses within metastatic niches. Sensory neuron–derived neurotransmitters and axon guidance cues have been shown to promote metastatic outgrowth across cancer types. For example, substance P released from sensory neurons enhances breast cancer metastasis via activation of the extracellular RNA–TLR7 signaling axis [83], while axon guidance molecule PlexinB3 expressed by sensory neurons facilitates metastatic progression in breast cancer models [84]. Neurotransmitter calcitonin gene‐related peptide (CGRP) has also been implicated in shaping immunosuppressive microenvironments in medullary thyroid cancer [85]. Furthermore, using retrograde neuronal tracing and transcriptional profiling, Thiel et al. demonstrated that pancreatic ductal adenocarcinoma (PDAC) actively reprograms peripheral neurons, and that combined neuronal denervation and chemotherapy synergistically suppress tumor growth [86].

Collectively, these studies highlight that neuroendocrine signaling, peripheral neural inputs, and the HPA–neuroimmune axis act as powerful systemic regulators of metastatic fate. By reshaping angiogenesis, inflammation, immune surveillance, and stromal architecture, these pathways can destabilize dormant states and promote metastatic reactivation. Thus, neuroendocrine and neuro‐immune pathways represent critical but underappreciated determinants of DTC awakening within metastatic microenvironments (Fig. 1F).

## 5 Therapeutic strategies targeting dormant DTCs

A major limitation of conventional cancer therapies is their reliance on targeting rapidly proliferating tumor cells, while sparing slow‐cycling or dormant disseminated tumor cells (DTCs) that can persist for years to decades and ultimately drive metastatic relapse. Importantly, as the duration and depth of dormancy vary widely across cancer types and organs, ranging from months to several decades, it further complicates both therapeutic timing and patient stratification. Advances in immunotherapy, together with improved understanding of dormant DTC biology and niche regulation, have paved the way for the development of therapeutic approaches that move beyond traditional cytotoxic chemotherapy. In lieu of these insights, strategies targeting cancer cell dormancy can be broadly categorized into three conceptual frameworks: (i) awakening dormant cells, (ii) enforcing or maintaining dormancy, and (iii) selectively eliminating dormant cells [87]. Each of these approaches presents distinct opportunities and limitations, particularly in the context of relapse risk prediction, treatment duration, and the challenge of assessing therapeutic efficacy in clinically undetectable disease.

**Fig. 2:**
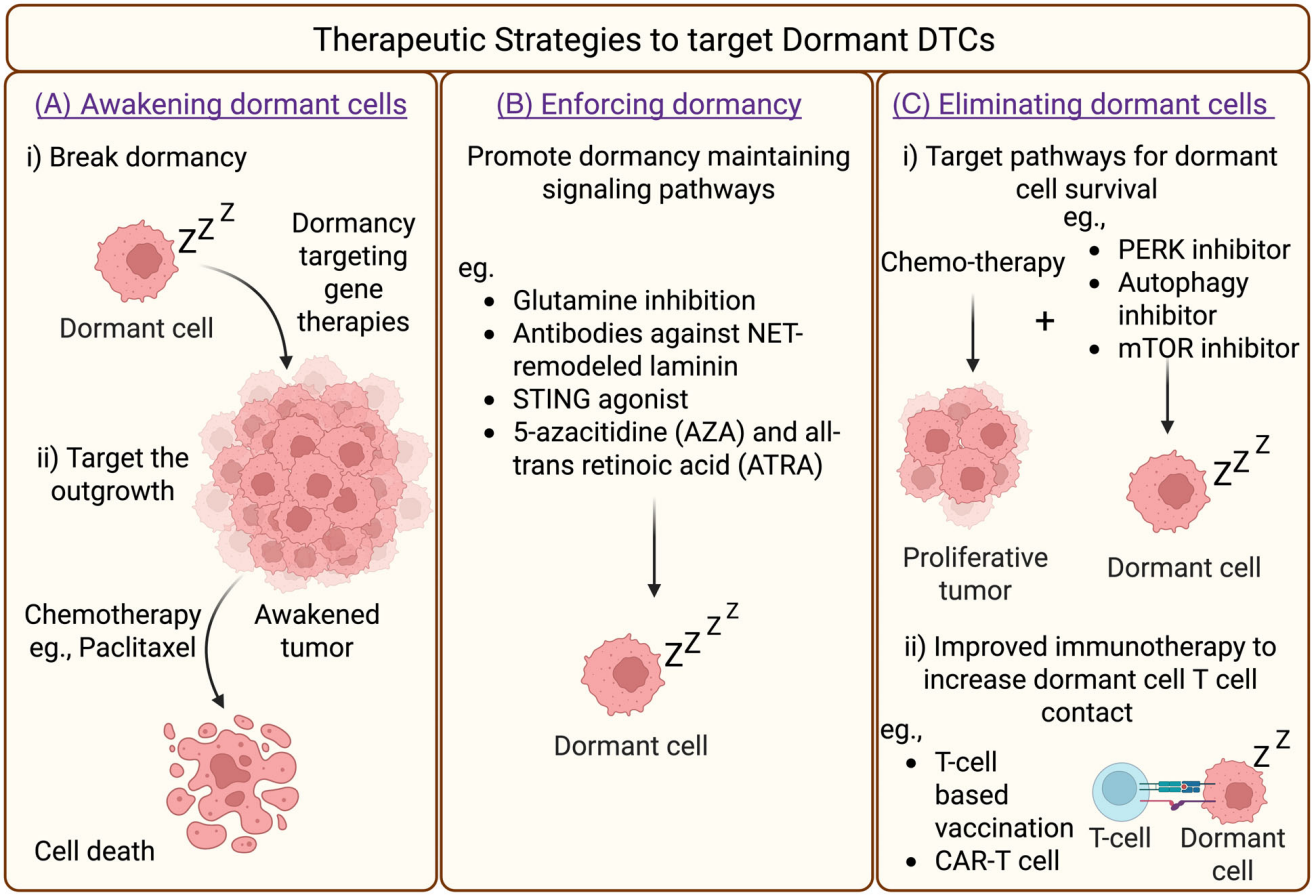
Therapeutic interventions to target dormant cancer cells: (A) awakening the tumor cells [88, 89]; (B) enforcing or maintaining dormancy [42, 76, 90, 91, 92] and (C) eliminating dormant tumor cells [66, 93, 94, 95, 96, 97, 98]. Graphical illustrations are created in Biorender.

- Awakening dormant tumor cells: This strategy aims to force dormant DTCs back into the cell cycle, thereby rendering them susceptible to conventional chemotherapies that preferentially target proliferating cells. Several studies have proposed pharmacological or microenvironmental perturbations to induce cell‐cycle re‐entry followed by cytotoxic treatment [88, 89]. Although conceptually appealing, this approach carries substantial risk as forced reactivation may inadvertently promote aggressive tumor behavior, enhance metastatic fitness, or allow incomplete elimination of reawakened cells, particularly in the absence of precise control over the timing and extent of proliferation. Moreover, given the heterogeneity of dormancy programs and the variable latency periods observed across cancer types, predicting which patients would benefit from awakening strategies remains challenging. As a result, the clinical efficacy of awakening followed by chemotherapy remains uncertain, and concerns persist regarding the potential for accelerating relapse rather than preventing it (Fig. 2A).
- Enforcing or maintaining dormancy: An alternative and increasingly attractive strategy focuses on maintaining DTCs in a dormant state by reinforcing tumor‐intrinsic quiescence programs or stabilizing dormancy‐supportive microenvironments (Fig. 2B). Multiple studies have demonstrated that components of the metastatic niche actively sustain dormancy and can be therapeutically exploited. For example, Albrengues et al. showed that inflammation‐induced neutrophil extracellular traps (NETs) remodel laminin in the lung metastatic niche and trigger awakening of dormant breast cancer DTCs; antibodies targeting NET‐remodeled laminin effectively prevented reactivation [76]. Similarly, NRF2‐driven metabolic reprogramming sensitizes dormant DTCs to glutaminase inhibition, thereby preventing dormancy escape [42]. STING agonists have also been shown to induce dormancy in lung cancer cells and reduce metastatic relapse [90]. Notably, a recent pilot clinical study demonstrated that combined treatment with 5‐azacitidine (AZA) and all‐trans retinoic acid (ATRA) induces tumor dormancy and delays clinical progression in prostate cancer patients with biochemical recurrence [91, 92].

Therapeutic strategies enforcing dormancy offer the advantage of limiting metastatic outgrowth without provoking tumor reactivation; however, they also raise important clinical concerns. In case that all dormant DTCs are not eliminated, and they may eventually acquire resistance, it can necessitate prolonged or lifelong treatment. Long‐term therapy increases the risk of toxicity, impacts patient quality of life, and complicates adherence to treatment protocols. Additionally, because dormant disease is clinically obscure, determining whether dormancy‐enforcing therapies are effective remains a major challenge. This underscores the clinical need for robust biomarkers to monitor residual disease and predict relapse risk.

iii Eliminating dormant tumor cells: Direct elimination of dormant DTCs represents the most definitive approach to preventing metastatic relapse and may complement conventional chemotherapy by eradicating residual reservoirs of disease (Fig. 2C). Several studies have identified vulnerabilities unique to dormant or slow‐cycling tumor cells. In non–small‐cell lung cancer (NSCLC), Cho et al. demonstrated that combined inhibition of COX‐2 and Src following chemotherapy reduces slow‐cycling cells and prevents disease progression [93]. Rehman et al. showed that colorectal cancer cells enter a diapause‐like drug‐tolerant persister state after chemotherapy and that targeting ULK1‐mediated autophagy in combination with CPT‐11 effectively eliminates these cells [94]. In breast cancer and NSCLC, inhibition of YAP/TEAD, ROCK, or fibronectin signaling induces apoptosis in dormant DTCs [95, 96].

Immunotherapy‐based approaches have also shown promise in overcoming the challenge posed by the scarcity and immune evasion of dormant DTCs. Goddard et al. demonstrated that T‐cell–based vaccination, adoptive T‐cell receptor transfer, and CAR T cell therapies can selectively eliminate metastatic breast cancer dormant DTCs in the lung metastatic niche [66]. Additionally, combination therapies have also been proven successful. Clinically, Calvo et al. reported that inhibition of the integrated stress response kinase PERK using the clinical‐grade inhibitor HC4 selectively targets ISR‐high, slow‐cycling dormant DTCs and significantly reduces metastasis; HC4 has completed phase I clinical trials in solid tumors (NCT04834778) [97, 98]. Additionally, DeMichele et al. showed that inhibition of mTOR signaling alone or in combination with autophagy inhibition (hydroxychloroquine) reduces residual tumor cell burden and improves recurrence‐free survival in breast cancer mouse models, findings that were successfully translated into a randomized phase II clinical trial (CLEVER), demonstrating substantial reductions in DTCs after treatment [98].

Collectively, these studies highlight the promise of integrating dormancy‐targeted therapies with established treatment modalities. However, successful clinical translation will require careful consideration of cancer‐type–specific dormancy kinetics, accurate prediction of relapse risk, and the development of sensitive tools to assess therapeutic efficacy in patients with clinically undetectable disease. Ultimately, combination strategies that balance efficacy, durability, and patient burden will be essential to effectively neutralize dormant cancer cells and prevent metastatic relapse.

## 6 Conclusions and perspectives

Metastatic dormancy represents a critical phase of cancer progression that profoundly influences long‐term patient outcomes. Dormant DTCs can persist for years to decades after primary tumor treatment and ultimately they serve as the reservoir for late metastatic relapse. There have been several studies that demonstrate that dormancy is not a passive state, but rather a dynamically regulated condition shaped by tumor‐intrinsic signaling, immune surveillance, and sustained interactions with the tumor microenvironment. However, despite major advances in single‐cell and spatial technologies, substantial gaps remain in our ability to detect, track, and functionally interrogate dormant DTCs *in vivo*, limiting both mechanistic understanding and clinical translation. We believe addressing these gaps will require improved preclinical models that faithfully recapitulate dormancy. Advanced imaging strategies and/or AI‐powered pathological detection of imminent cancer relapse could enable earlier therapeutic intervention. Additionally, biomarkers that can distinguish dormant, awakening, and irreversibly quiescent tumor cell states.

While dormancy arises through diverse, immune‐independent mechanisms, immune surveillance plays a pivotal role in determining the persistence and clinical relevance of dormant DTCs. Therefore, in depth investigation of immune–dormant DTC interactions in an organ specific manner will be essential to determine whether and how the immune system can be leveraged to clear dormant cells. Focusing on immune‐specific adaptations may therefore enable selective targeting of high‐risk dormant cells without broadly disrupting tissue homeostasis. However, it remains unclear whether long‐term clinical benefit will be achieved through complete eradication of dormant DTCs, therapeutic reinforcement of dormancy, or a cancer stage‐dependent combination of both strategies. Importantly, interventions must be carefully designed to avoid inadvertently promoting metastatic awakening. In conclusion, a deeper understanding of how immune regulation intersects with dormancy programs across organs and disease stages will be essential for transforming metastatic dormancy from a clinical uncertainty into a tractable therapeutic opportunity.

## Conflict of interest

J.J.B.C. is a consultant for HTL Biotechnology.

## Author contributions

KT performed conceptualization, literature review, writing of the original draft, and visualization. JJBC provided supervision, critical editing, revision, and funding acquisition. All authors contributed to manuscript revision and approved the final version.

## Data Availability

Data sharing is not applicable to this article as no new data were created or analyzed in this study.
